# Diagnostic Value of ^18^F-FDG PET/CT in Patients with Carcinoma of Unknown Primary

**DOI:** 10.4274/mirt.64426

**Published:** 2018-10-09

**Authors:** Arzu Cengiz, Sibel Göksel, Yakup Yürekli

**Affiliations:** 1 Adnan Menderes University Faculty of Medicine, Department of Nuclear Medicine, Aydın, Turkey

**Keywords:** Fluorodeoxyglucose, positron emission tomography/computed tomography, metastasis, unknown primary neoplasms

## Abstract

**Objective:**

The aim of this study is to investigate the clinical role of ^18^F-fluorodeoxyglucose (^18^F-FDG) positron emission tomography/computed tomography (PET/CT) in patients with carcinoma of unknown primary (CUP).

**Methods:**

One hundred twenty one patients with a diagnosis of CUP who underwent whole body ^18^F-FDG PET/CT imaging were included in this retrospective study. The final diagnoses were confirmed either histopathologically or by clinical follow-up.

**Results:**

The ^18^F-FDG-PET/CT successfully detected the primary tumor in 59 out of 121 (49%) patients. The most common primary tumor as detected by ^18^F-FDG PET/CT was lung cancer (n=31). In a patient, two primary tumors (colon and prostate) were detected on PET/CT imaging. Bone marrow biopsy revealed prostate cancer in this patient and the colon cancer was accepted as a synchronous second primary tumor. ^18^F-FDG PET/CT findings were false-positive in 11 patients. ^18^F-FDG PET/CT could not detect any primary lesion in 51 patients, whose conventional work-up detected a primary tumor in 11 and thus considered as false-negative. The sensitivity, specificity rate and accuracy of ^18^F-FDG PET/CT in detection of primary tumor were identified as 84%, 78% and 82%, respectively.

**Conclusion:**

Whole body ^18^F-FDG PET/CT is an effective method for detecting the primary tumor in patients with CUP. In addition to detecting the primary tumor, it can also help determine disease extent and contribute to patient management.

## Abstract (tr)

**Amaç:**

Bu çalışmanın amacı, primeri bilinmeyen kanserlerde (PBK) ^18^F-florodeoksiglukoz (^18^F-FDG) pozitron emisyon tomografi/bilgisayarlı tomografinin (PET/BT) klinik rolünü araştırmaktır.

**Yöntem:**

Bu retrospektif çalışmaya PBK tanısıyla tüm vücut ^18^F-FDG PET/BT yapılan 121 hasta dahil edildi. Sonuç tanı histopatolojik olarak veya klinik izlemle doğrulandı.

**Bulgular:**

^18^F-FDG PET/BT, 121 hastanın 59’unda (49%) primer tümörü saptadı. PET/BT ile en çok saptanan tümör akciğer kanseri idi (n=31). Bir hastada iki primer tümör saptandı (kolon ve prostat). Bu hastada kemik iliği biyopsisi prostat kanseri metastazını gösterdiği için kolon kanseri senkron ikinci primer olarak kabul edildi. ^18^F-FDG PET/BT bulguları 11 hastada yanlış pozitif idi. PET/BT ile herhangi bir lezyon saptanmayan 51 hastanın 11 tanesinde konvansiyonel tetkiklerle primer tümör saptandı ve bu hastalar yanlış negatif olarak değerlendirildi. Primer tümör saptanmasında ^18^F-FDG PET/BT ile duyarlılık, özgüllük ve doğruluk sırasıyla %84, %78 ve %82 idi.

**Sonuç:**

PBK olan hastalarda primer tümörün saptanmasında tüm vücut ^18^F-FDG PET/BT etkin bir yöntemdir. Primer tümör saptanması yanında hastalığın yaygınlığını da belirleyerek hastaların izlemine katkıda bulunur.

## Introduction

Carcinoma of unknown primary (CUP) refers to the presence of metastatic disease for which the site of the primary lesion remains unidentified after conventional diagnostic procedures. CUP accounts for approximately 2.3-4.2% of cancer in both men and women (1,2). The mean survival is between 3-11 months, and only 25% of patients survive over one year (3,4). Several studies have shown that survival of patients in whom the primary tumor has been detected was higher than that of patients in whom the primary tumor has remained unknown (5,6). Various radiologic methods and serum tumor markers can be used for primary tumor detection. However, the primary tumor could be detected in less than 20% of patients with CUP (1). Although spontaneous regression or immune-mediated destruction of primary tumor or the small size of a primary tumor may be an explanation, it is not yet fully understood why primary tumors remain undetected (2,7,8).

Several studies reported that ^18^F-fluorodeoxyglucose (FDG) positron emission tomography/computed tomography (PET/CT) has higher sensitivity than other imaging methods for detection of the primary tumor (9,10,11).

The aim of this retrospective study is to evaluate to primary tumor detection efficiency of ^18^F-FDG PET/CT in patients with CUP.

## Materials and Methods

### Patient Population

All patients who have been referred to our department for ^18^F-FDG PET/CT with a diagnosis of CUP from April 2013 to March 2016 were retrospectively evaluated. Patients who had inadequate medical records or irregular clinical follow-up data and who had chemotherapy before imaging were excluded. 121 patients (79 men, 42 women, age range 30-86 years, mean 63±12 years) were analyzed finally in the study. Ninety five out of 121 patients were proved to have metastases histopathologically and 26 patients had highly suspicious metastases by conventional imaging [8 patients with multiple lung metastases detected by CT, 10 patients with multiple bone metastases detected by scintigraphy and/or magnetic resonance imaging (MRI), 5 patients with multiple liver metastases by MRI and/or US, and 3 patients with brain metastases detected by MRI]. Locations of the metastatic foci that have been proven histologically were as follows; 36 in lymph nodes, (21 cervical, 6 supraclavicular, 4 axillary, 2 mediastinal, 2 inguinal, 1 retroperitoneal), 19 in liver, 13 in bone, 6 in brain, 3 in soft tissue, 1 in adrenal gland, 1 in lung, 9 patients had peritoneal implants or malignant ascites, 6 patients had malignant pleural effusion and 1 patient had malignant pericardial effusion.

The study were approved by the Adnan Menderes University of Local Ethics Committee (protocol number: 2017/1043).

### ^18^F-FDG PET/CT Imaging

All patients underwent ^18^F-FDG PET/CT imaging after 6-8 hours of fasting. Before injection of ^18^F-FDG, the medical history, weight and blood sugar level of the patients were recorded. All patients’ blood sugar levels were less than 180 mg/dL prior to imaging. Oral contrast was given to all patients. After intravenous administration of 270-370 MBq of ^18^F-FDG, patients rested in a quiet room. Imaging was performed after a resting period of 60 minutes with (Siemens Biograph mCT 20 Excel) PET/CT scanner. Images were acquired from the head to the feet. The CT transmission scan was acquired with 140 kVp and 110 mA and 3 mm slice thickness. PET scan was acquired at 2-4 min per bed position. ^18^F-FDG PET/CT images were evaluated both visually and semi-quantitatively by two nuclear medicine physicians. Abnormal ^18^F-FDG uptake (SUV_max_ ≥2.5) with an anatomical correlation in any tissue or organ other than the metastases sites was considered as the primary site. The final results were confirmed either histopathologically or by clinical follow up including other imaging methods.

### Data Analysis and Statistical Evaluation

The final diagnosis was considered true-positive (TP) when ^18^F-FDG PET/CT detected the primary tumor and it was confirmed histopathologically and/or by clinical follow up. If it was not confirmed to be malignant histopathologically then the result was considered as false-positive (FP). If ^18^F-FDG PET/CT could not detect the primary tumor and it remained unknown in follow up, the result was considered true-negative (TN). When ^18^F-FDG PET/CT did not suggest any primary tumor but it was diagnosed with conventional work-up or in the patient’s follow-up, the result was considered as false-negative (FN).

Sensitivity, specificity rates and accuracy were calculated using standard statistical formulas:

Sensitivity=TP/(TP+FN), Specificity=TN/(TN+FP), Accuracy=(TP+TN)/(TP+FP+TN+FN).

## Results

**Figure 1:**
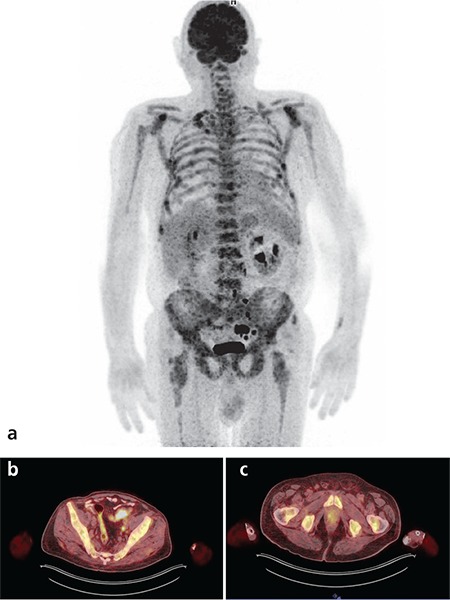
^18^F-FDG PET/CT images of a 72-year-old male patient with bone metastasis proven histopathologically. MIP (a), fusion (b and c) images showed hyper-metabolic focus in the prostate and wall-thickness on descending colon with pathologically increased ^18^F-FDG uptake, which were later confirmed as prostate adenocarcinoma and colon adenocarcinoma by histopathology

Primary tumors were correctly detected in 59 of 121 patients (49%) by ^18^F-FDG PET/CT whole body imaging. The primary tumor locations were as follows; lung (n=31), breast (n=3), stomach (n=1), colon (n=4), pancreas (n=2), ovary (n=3), prostate (n=4), liver (n=2), endometrium (n=1), skin (n=2), thyroid (n=2), larynx (n=1), hypopharynx (n=1), salivary gland (n=1) and bone marrow (multiple myeloma; n=1). In a patient, two primary tumors (colon and prostate) were detected by PET/CT imaging both of which were confirmed histopathologically (Figure 1). In this patient, the bone marrow biopsy revealed metastatic prostate carcinoma thus the colon carcinoma was accepted as a synchronous second primary tumor. Fifty-nine TP results were selected for statistical evaluation. The SUV_max _of the hyper-metabolic lesions were between 3 to 27 (mean 11.57±6.1). TP results are reported in Table 1.

The sensitivity, specificity rates and accuracy of ^18^F-FDG PET/CT in detection of primary tumor were identified as 84%, 78% and 82%, respectively. When 36 patients with lymph node metastases were evaluated separately, primary tumors were correctly identified in 14 out of 36 patients. In these cases, the sensitivity, specificity and accuracy were calculated as 66%, 75% and 70%, respectively.

**Figure 2:**
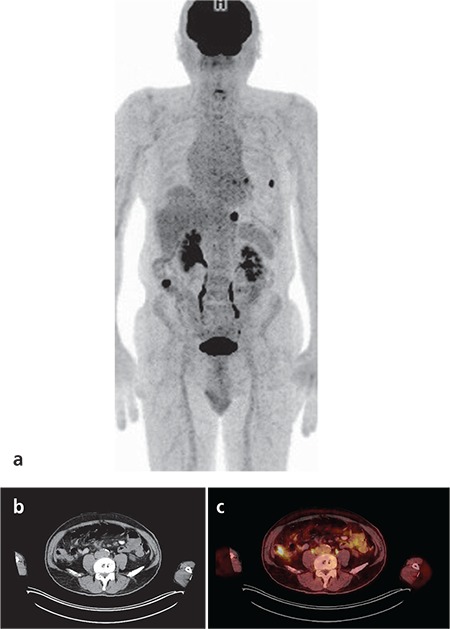
MIP (a), CT (b) and fusion (c) ^18^F-FDG PET/CT images of a 64 yearold male patient. Cervical lymph node biopsy revealed adenocarcinoma metastasis. On PET/CT imaging, there were multiple hyper-metabolic mediastinal lymph nodes and mild hyper-metabolic infiltrations in both lungs suggesting infection. PET/CT imaging also demonstrates wall thickness on the ascending colon with abnormally increased ^18^F-FDG uptake (SUV_max_: 5.0), which was interpreted as a primary tumor. The histopathology examination revealed a hyperplastic polyp. The ^18^F-FDG PET/CT result was false-positive

There were eleven patients in whom primary tumors were reported incorrectly by ^18^F-FDG PET/CT imaging. These results were accepted as false-positive (Table 2). A false-positive case is presented in Figure 2.

The primary tumor could not be identified in 51 (42%) patients. Forty of these patients were TN. The remaining 11 patients, ^18^F-FDG PET/CT did not detect any lesion but the primary tumors were detected during clinical follow-up (mean 6.8 months, range: 2-30 months). These FN results are listed in Table 3.

Additional distant metastases were detected in 45 out of 59 (76%) patients whose primary tumors were detected correctly by ^18^F-FDG PET/CT. In patients with only lymph node metastases, additional solid organ metastases were detected in 5 patients out of 36 (14%) with PET/CT imaging.

## Discussion

CT and MRI have been the imaging methods of choice in clinical practice in patients with CUP. Although they detect anatomical abnormalities with pathologic contrast enhancement, small or non-enhancing lesions can be overlooked (1). ^18^F-FDG PET/CT is gaining acceptance as an imaging method to be used in the management of patients with CUP. Small lesions can be detected with higher sensitivity due to its high lesion-to-background contrast. Several studies reported that ^18^F-FDG PET/CT is more sensitive than CT and MRI in the imaging of CUP. In a study, Gutzeit et al. (12) have shown that CT alone indicated a primary tumor in only 8 of 45 patients (18%) while ^18^F-FDG PET/CT detected the primary site in 15 of 45 patients (33%). In another study, Roh et al. (13) have reported that the sensitivity rate of ^18^F-FDG PET/CT (87.5%) was significantly higher than that of CT (43.7%) for the primary tumor in patients with cervical metastases from unknown origin. In several studies, primary tumor detection rate ranged between 24.5-53% for ^18^F-FDG PET/CT in patients with CUP (11,14,15,16). Consistent with the literature, in this study, primary tumors were correctly detected in 59 of 121 patients (49%) by ^18^F-FDG PET/CT whole body imaging. The sensitivity, specificity rates and accuracy of ^18^F-FDG PET/CT in detection of primary tumor were identified as 84%, 78% and 82%, respectively. Han et al. (17) reported the sensitivity, specificity and accuracy of ^18^F-FDG PET/CT in patients with CUP as 91.5%, 85.2% and 88.3%, respectively. In another study, the sensitivity, specificity and accuracy of ^18^F-FDG PET/CT in detection of primary tumor were reported as 80%, 74% and 78%, respectively (18). In our study, ^18^F-FDG PET/CT was the first imaging method used for detecting the primary in majority of the patients. Although the role of ^18^F-FDG PET/CT as the first line imaging of patients with CUP is yet to be established, it has significant advantages. Whole body imaging demonstrates disease extent in addition to detection of the primary tumor, eliminates the need for further imaging and other invasive procedures. Thus, it prevents delay in starting appropriate treatment (19,20).

Lung, oropharyngeal and pancreatic cancers were reported to be most common primary tumors in patients with CUP (21). In our study, lung (52%) and colon (8%) were the most common sites for primary tumors. Colorectal cancer is the third most common cancer in women and the fourth in men in our country (22). Although there were 21 patients with cervical lymph node metastases in our study, we detected 5 head and neck tumors as true-positive.

The most important limitation of ^18^F-FDG PET/CT is that it’s not a specific tumor imaging technique. Inflammatory lesions or benign tumors with high tracer uptake are the most common causes of false-positive results. In our study, there were eleven false-positive results related to benign tumors or inflammation. In a meta-analysis, authors reported that oropharynx and the lung are the two most common locations of false-positive ^18^F-FDG PET/CT results (21). Inflammatory lesions, pulmonary infarction and emboli have been reported as etiologies for false-positive results in the lung (2,12). In this study, 3 out of the 11 false-positive results were detected in the lung. Pulmonary alveolar proteinosis, hamartoma and inflammation were the final diagnosis in these patients. PET/CT diagnosed a false-positive colon cancer in three patients. The final diagnoses were polyps in two patients and diverticulitis in one patient, that were confirmed histopathologically. In a study, the authors concluded that if ^18^F-FDG PET/CT findings are positive, a confirmatory biopsy is necessary due to false-positive results (23).

In our study, ^18^F-FDG PET/CT could not detect the primary tumor in 42% of patients. Primary tumors were detected on follow-up in 11 out of 51 patients and were considered as FN. Small and low grade tumors with low ^18^F-FDG uptake may result in FN findings. Breast and oropharynx are the most common sites for FN ^18^F-FDG PET/CT imaging (21). In this study, a small primary breast cancer was detected by MRI and was histopathologically diagnosed as invasive ductal cancer following a FN ^18^F-FDG PET/CT imaging. In four patients, lung tumors with low ^18^F-FDG avidity caused FN results.

Whole body ^18^F-FDG PET/CT is also useful in detecting the extent of metastatic disease which may have important implications for clinical management. It is especially important in patients with initial lymph node metastases (2,24). We showed additional solid organ metastases in 5 out of 36 (14%) patients with CUP who presented with lymph node metastases on PET/CT imaging.

## Conclusion

Whole body ^18^F-FDG PET/CT is an effective method for detecting the primary tumors in patients with CUP. Additionally, it can also determine disease extent and contribute significantly to clinical patient management.

## Figures and Tables

**Table 1:**
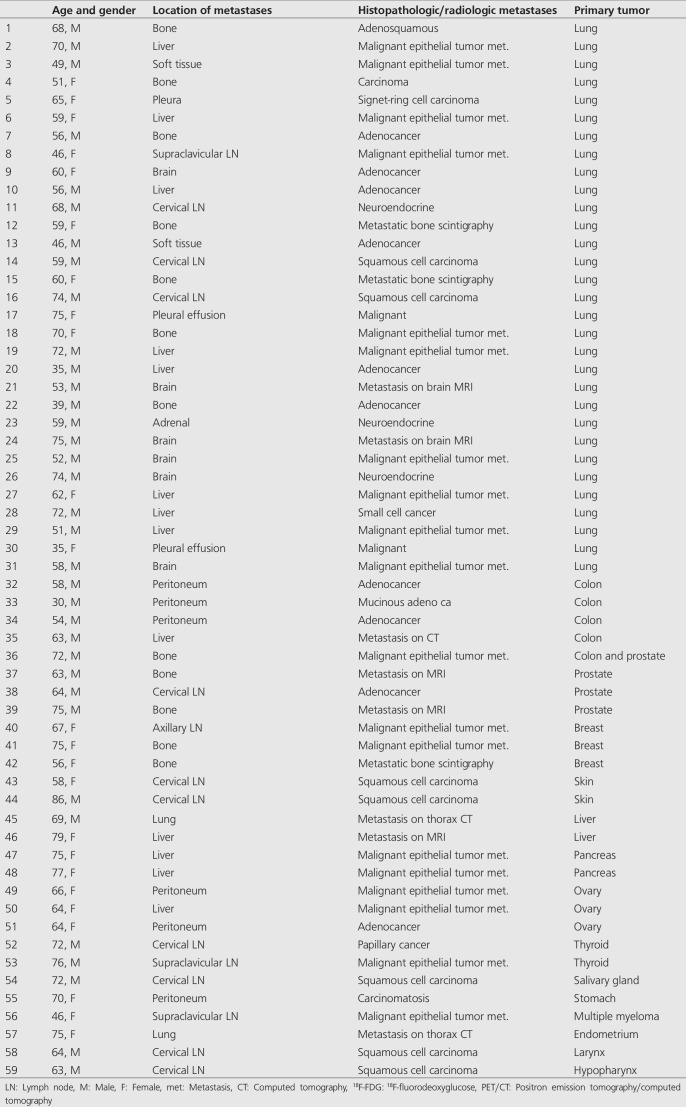
There were fifty-nine patients with sixty true-positive results diagnosed by ^18^F-fluorodeoxyglucose positron emission tomography/computed tomography

**Table 2:**
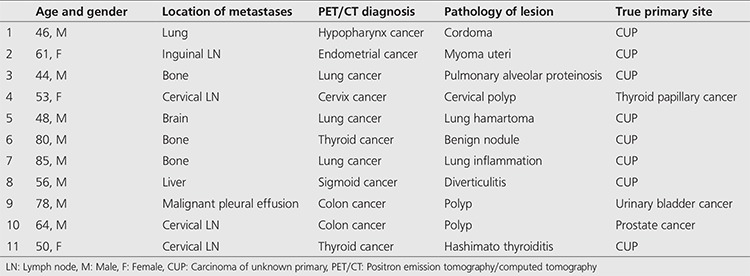
The eleven false-positive results diagnosed by ^18^F-fluorodeoxyglucose positron emission tomography/computed tomography

**Table 3:**
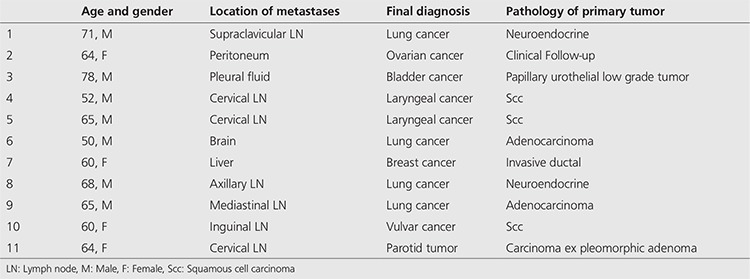
False-negative results of ^18^F-fluorodeoxyglucose positron emission tomography/computed tomography in patients with carcinoma of unknown primary
